# Infected Frontal Sinus Mucocele Mimicking a Cutaneous Abscess

**DOI:** 10.7759/cureus.115941

**Published:** 2026-09-08

**Authors:** Laura G Ferretti, Herve Zender, Chiheb Said

**Affiliations:** 1 Department of Internal Medicine, Neuchâtel Hospital Network, La Chaux-de-Fonds, CHE; 2 Department of Acute Medicine, University Hospital of Geneva, Geneva, CHE; 3 Faculty of Medicine, University of Geneva, Geneva, CHE; 4 School of Medicine, University Hospital of Bern, Bern, CHE

**Keywords:** bony erosion, cutaneous abscess, diagnostic pitfall, endoscopic sinus surgery, frontal sinus mucocele, frontal swelling, periorbital cellulitis

## Abstract

Frontal sinus mucoceles are benign expansile lesions that may progressively erode the surrounding bone and extend toward the orbit or intracranial compartment. When expansion occurs anteriorly, however, they may present as an apparently superficial forehead swelling and be mistaken for a cutaneous abscess.

A 77-year-old woman with chronic sinusitis developed recurrent left paramedian frontal swelling over several months, with only transient improvement following antibiotic treatment. The lesion was initially interpreted as a cutaneous abscess and incised in an outpatient setting. Clear, nonpurulent fluid was obtained instead of pus. Two days later, she presented with persistent frontal inflammation and newly developed left periorbital cellulitis. Despite the extent of the disease, she remained afebrile and had no headache, neurological symptoms, diplopia, or visual impairment. CT and MRI demonstrated a left frontal sinus mucocele with erosion of both the anterior and posterior frontal sinus tables, in close proximity to the superomedial orbit and dura mater. After control of the acute inflammatory process, the mucocele was treated with endoscopic endonasal marsupialization through a Draf IIc frontal sinusotomy. Complete clinical resolution was achieved, with no documented recurrence during one year of follow-up.

This case illustrates an important diagnostic pitfall: the superficial appearance of a frontal swelling may conceal extensive underlying sinus disease and bony erosion. Recurrent paramedian frontal swelling, transient responses to antibiotics, clear rather than purulent drainage, or associated periorbital inflammation should prompt CT or MRI before any further incision.

## Introduction

Paranasal sinus mucoceles are benign, epithelium-lined, mucus-filled expansile lesions that develop as a result of obstruction of sinus drainage. Progressive accumulation of secretions can produce pressure-induced remodeling and erosion of the surrounding bone. Frontal and frontoethmoidal locations are clinically important because expansion may extend toward the forehead, orbit, or intracranial compartment [1,2]. Orbital involvement may manifest as proptosis, diplopia, impaired ocular motility, or visual impairment, whereas intracranial extension may present with severe headache, meningeal symptoms, altered mental status, seizures, or focal neurological deficits [1,2]. Anteriorly expanding frontal mucoceles may instead appear as a subcutaneous forehead mass and mimic a primary cutaneous lesion [3].

Secondary infection may further obscure the diagnosis by producing erythema, tenderness, periorbital inflammatory changes, and temporary improvement with antibiotic therapy. Presentation as a presumed cutaneous abscess is uncommon and may lead to inappropriate incision and delayed diagnosis. Recurrent paramedian frontal swelling, recurrence shortly after antibiotic discontinuation, clear rather than purulent fluid after incision, or associated periorbital findings should raise suspicion for an underlying frontal sinus process. Simple cutaneous drainage does not address the obstructed sinus and may be hazardous when the sinus walls are eroded. CT is central to evaluating bony anatomy, whereas MRI helps characterize the lesion and assess its relationship to the orbit, dura mater, and intracranial structures [1,4,5]. We report an infected frontal sinus mucocele presenting as recurrent frontal swelling with periorbital cellulitis after incision as a presumed cutaneous abscess, illustrating an uncommon presentation and an important diagnostic pitfall in the evaluation of atypical frontal swellings.

## Case presentation

The patient was a 77-year-old woman with a medical history of chronic sinusitis, hypertension, sinus tachycardia, and total hip arthroplasty. For several months, she had experienced recurrent left paramedian frontal swelling, with transient improvement following previous outpatient antibiotic courses and recurrence shortly after discontinuation of treatment. The agents, doses, and durations of these earlier courses, as well as the precise extent and duration of the clinical responses, were unavailable. The most recent treatment consisted of approximately nine days of clindamycin, at an undocumented dose, without clinical improvement.

The lesion was initially interpreted as a cutaneous abscess and was incised in an outpatient setting. The procedure yielded clear, nonpurulent fluid rather than pus, and no sample was submitted for biochemical or microbiological analysis. Two days later, she presented to our emergency department because of persistent frontal inflammation and new-onset left periorbital cellulitis.

On examination, she was afebrile. There was an indurated, erythematous, and tender swelling involving the left paramedian frontal and interbrow regions, with inflammatory changes extending to the left periorbital tissues (Figure 1).

**Figure 1:**
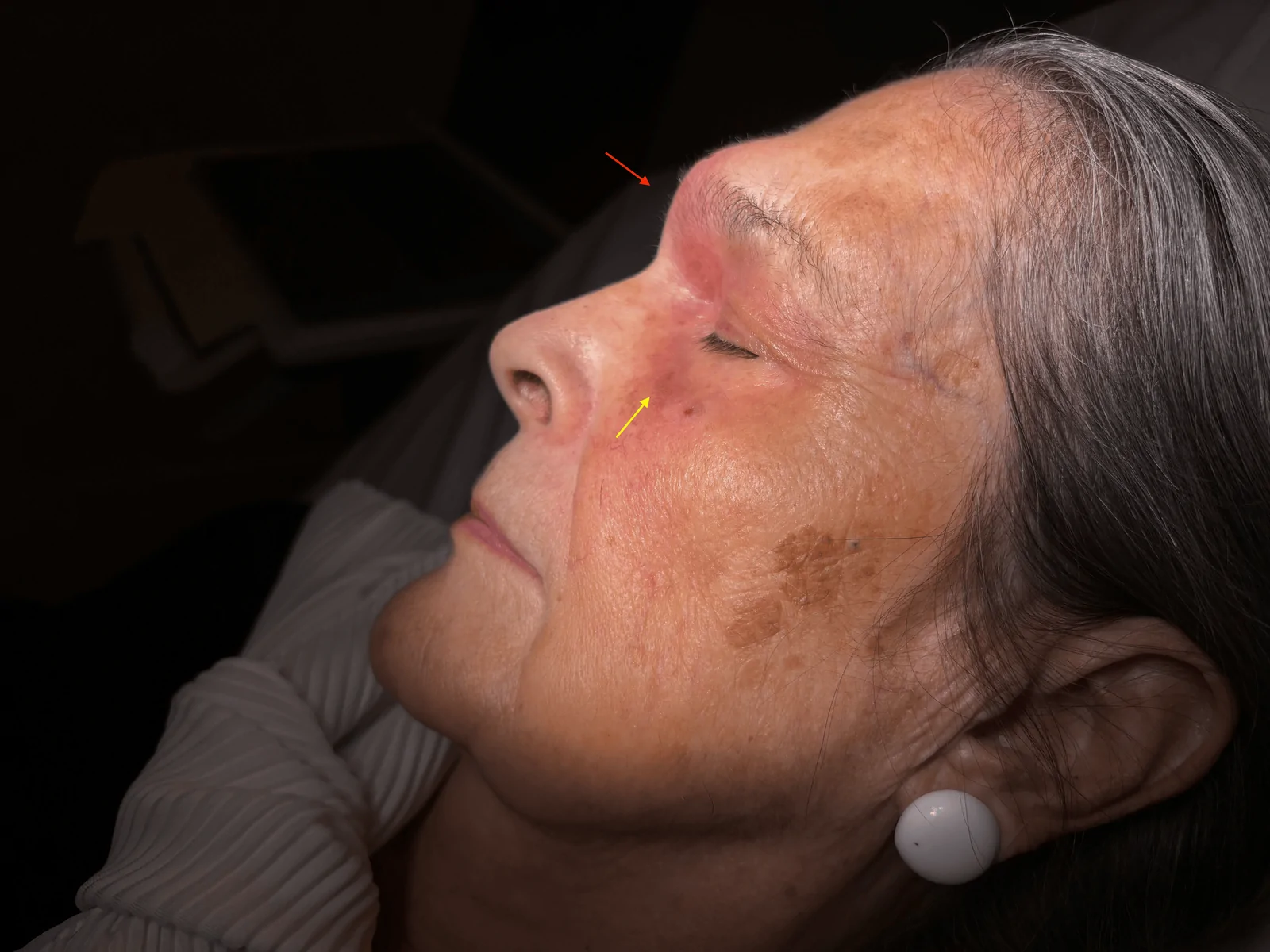
Left frontal swelling with associated periorbital inflammatory changes Clinical photograph obtained at presentation to the emergency department showing an indurated and erythematous left paramedian frontal swelling, with inflammatory changes extending to the left periorbital tissues (arrows)

Ocular motility was preserved, and she reported no diplopia or visual impairment. She also denied chills, headache, or neurological symptoms. The nasal mucosa was unremarkable, and no inflammatory changes were observed in the oral cavity. Apart from the documented absence of fever, complete admission vital signs, complete blood count, and C-reactive protein values were unavailable in the records reviewed for this report.

CT of the head and paranasal sinuses demonstrated a left frontal sinus mucocele with erosion of both the anterior and posterior frontal sinus tables (Figure 2).

**Figure 2:**
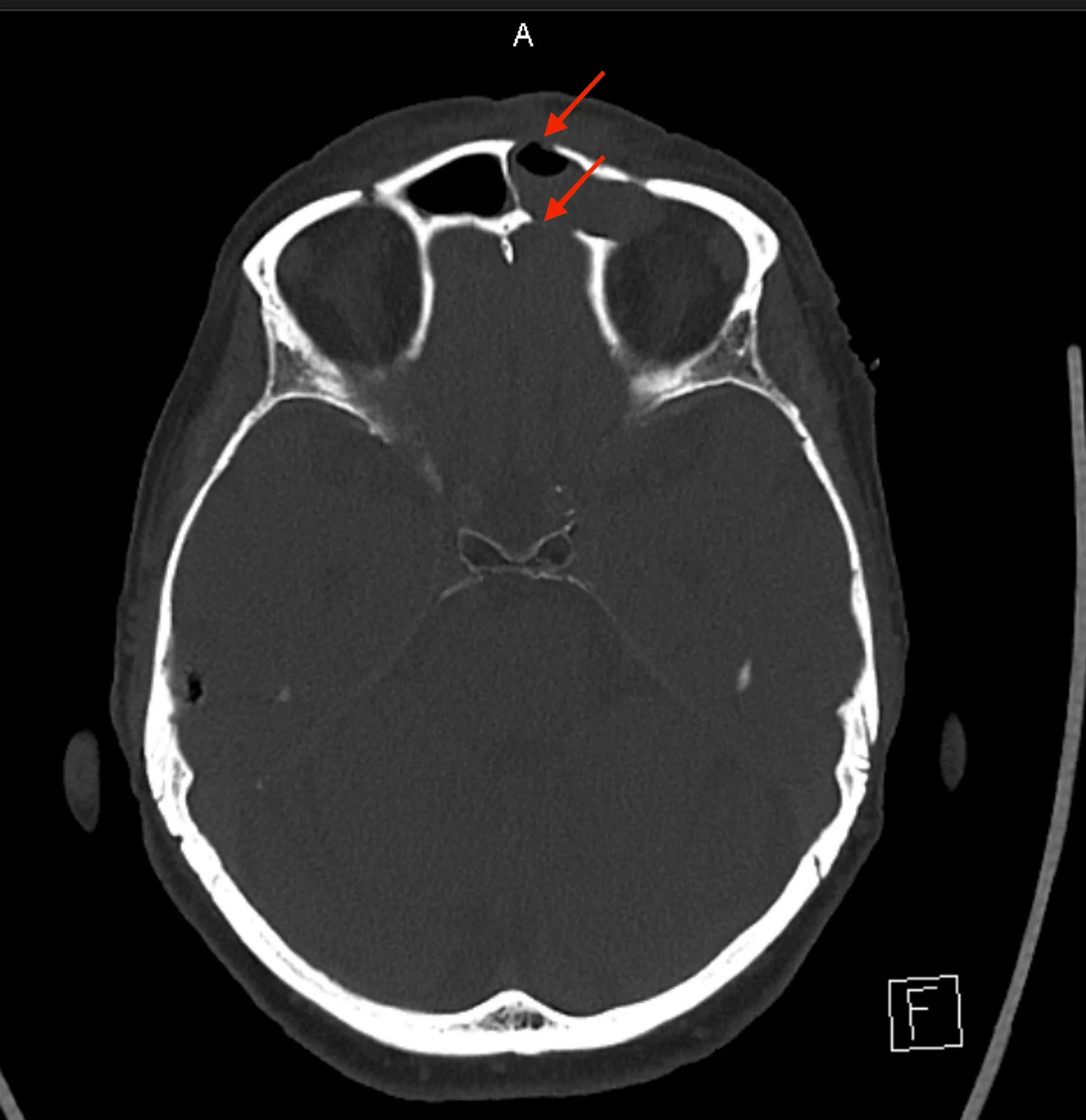
CT showing left frontal sinus mucocele with anterior and posterior table erosion Axial CT image in bone window demonstrating a left frontal sinus mucocele measuring approximately 24 × 8 mm in the axial plane, with erosion of the anterior table (upper arrow) and posterior table (lower arrow) CT: computed tomography

Following assessment and CT imaging in our emergency department, the patient was referred to a university otolaryngology center. She received intravenous cefuroxime at a total dose of 2 g/24 h for two days. A single 8-mg intravenous dose of dexamethasone was administered concomitantly as adjunctive therapy to reduce the marked periorbital inflammatory edema; corticosteroid therapy was limited to this single dose. During this initial hospital admission, a repeat incision and drainage procedure of the frontal swelling was performed under local anesthesia. Culture of the fluid obtained during this second procedure grew *Streptococcus anginosus*, which was resistant to clindamycin. On hospital day two, intravenous cefuroxime was switched to oral cefuroxime 500 mg every 12 hours and continued for eight days. Following clinical improvement, she was discharged after a four-day hospitalization.

Thirteen days after initiation of antibiotic therapy, MRI further characterized the lesion and demonstrated its close relationship to the adjacent orbital structures and dura mater (Figures 3-5). Neither CT nor MRI demonstrated a separately identifiable subperiosteal abscess, and no radiological findings specifically diagnostic of frontal bone osteomyelitis were documented.

**Figure 3:**
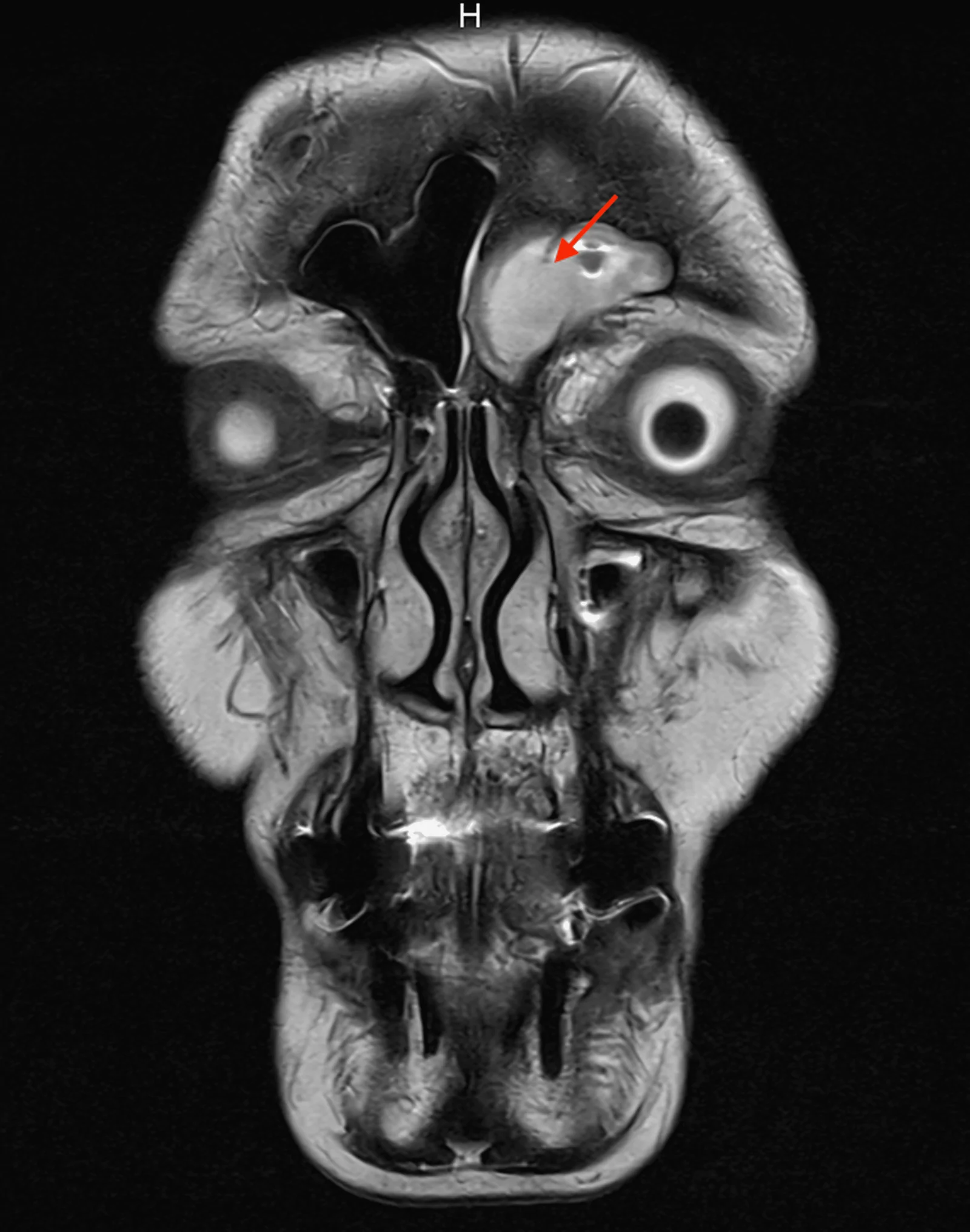
Coronal T2-weighted MRI of the left frontal sinus mucocele Coronal T2-weighted turbo spin-echo MRI scan acquired with a 2.5-mm slice thickness, showing the T2-hyperintense left frontal sinus mucocele and its close relationship to the superomedial left orbit and adjacent frontal structures (arrow) MRI: magnetic resonance imaging

**Figure 4:**
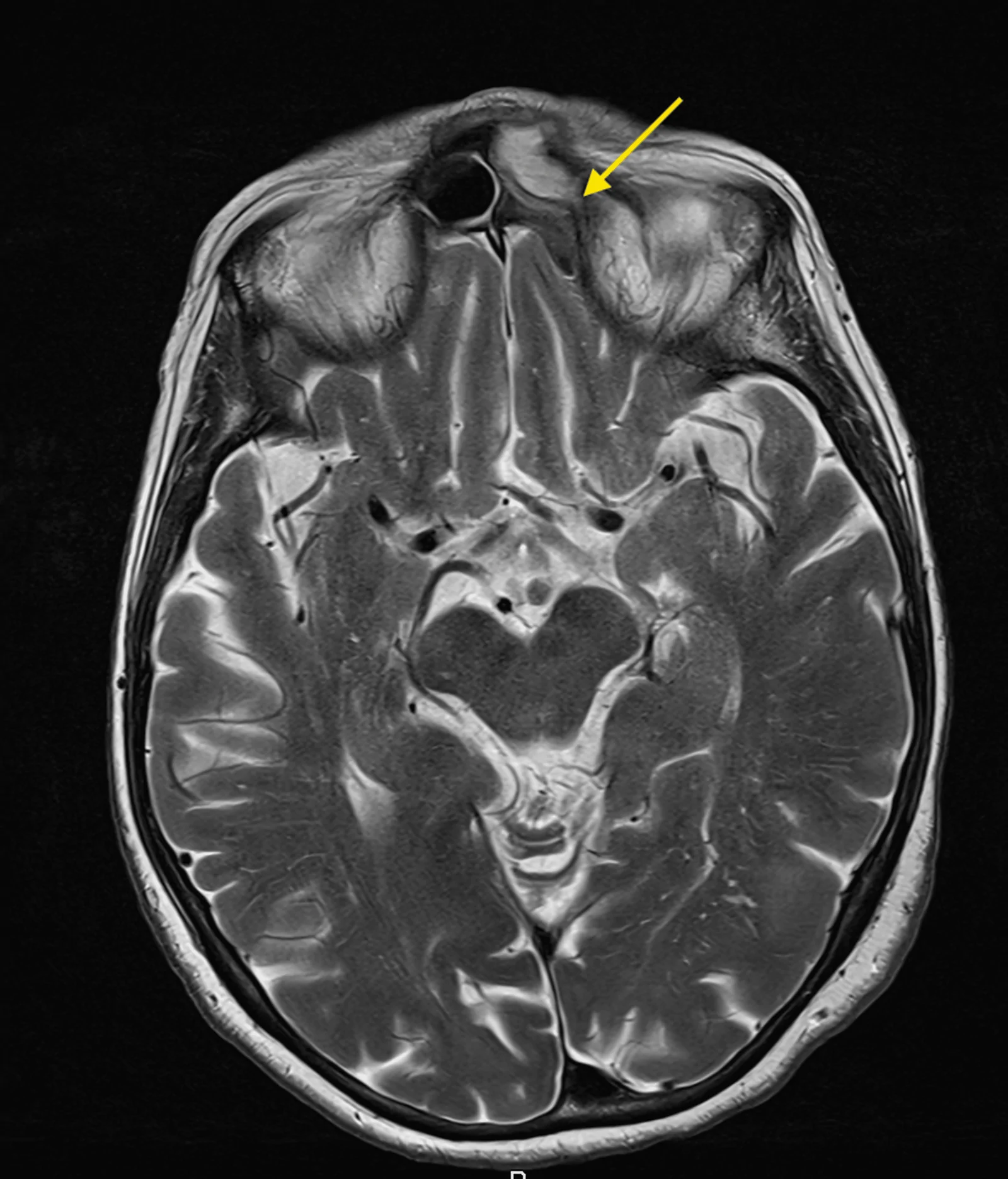
Axial T2-weighted MRI showing the orbital relationship of the left frontal sinus mucocele Axial T2-weighted MRI scan demonstrating the close relationship between the left frontal sinus mucocele and the superomedial aspect of the left orbit. The arrow indicates the interface between the lesion and the adjacent periorbita MRI: magnetic resonance imaging

**Figure 5:**
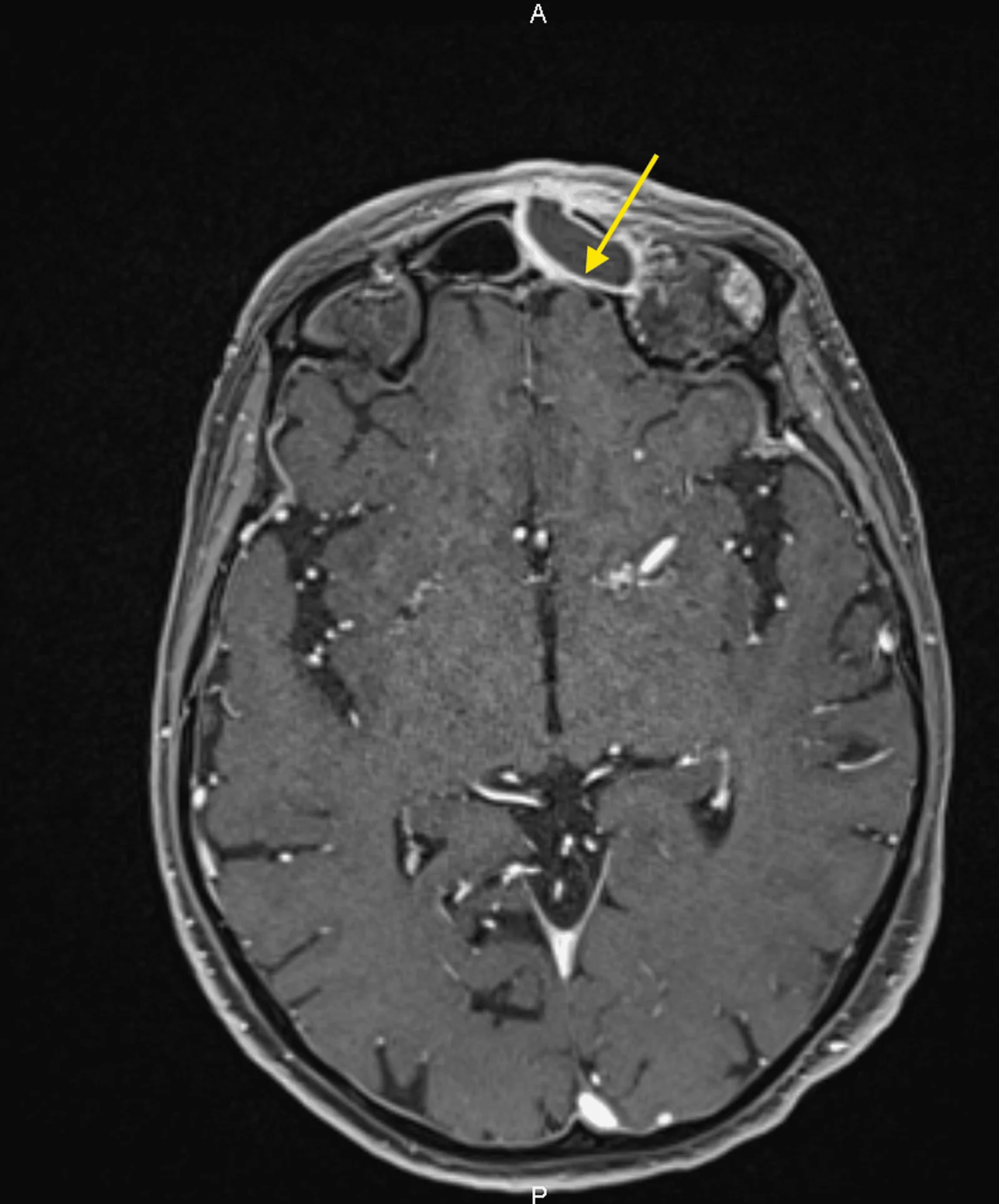
Axial contrast-enhanced T1-weighted MRI showing the posterior relationship of the left frontal sinus mucocele Axial gadolinium-enhanced, fat-suppressed T1-weighted VIBE MRI scan demonstrating peripheral enhancement of the left frontal sinus mucocele. The arrow indicates the posterior margin of the lesion adjacent to the posterior frontal sinus table and dura mater MRI: magnetic resonance imaging; VIBE: volumetric interpolated breath-hold examination

Twenty-one days after initiation of antibiotic therapy, following resolution of the acute inflammatory changes, the patient underwent endoscopic endonasal surgery consisting of an extended left frontal sinusotomy (Draf IIc), anterior ethmoidectomy, maxillary antrostomy, septoplasty to facilitate surgical access, and marsupialization of the mucocele. The left frontal drainage pathway was identified and opened, releasing the mucocele contents, and the opening was subsequently enlarged to achieve wide marsupialization of the lesion. An intraoperative bacteriological swab obtained directly from the mucocele contents showed no growth.

The histopathological specimen consisted of all fragmented surgical material submitted from the left frontal sinus and mucocele region during marsupialization; no specific portion of the cyst wall was separately identified or anatomically oriented. The entire 6 cm³ of material received by the pathology laboratory was processed. Histopathological analysis demonstrated fragments of respiratory mucosa with mild-to-moderate chronic inflammation and edema of the lamina propria, together with mucus and bone fragments. Although bone fragments were present, the pathology report did not describe histological features of osteomyelitis within the bone fragments examined. No evidence of malignancy was identified. The final histopathological diagnosis was chronic inflammation of the sinonasal mucosa.

The patient was discharged on the second postoperative day and completed an additional seven-day course of oral cefuroxime. At clinical follow-up on postoperative day seven, the frontal and periorbital inflammatory changes had completely resolved. Follow-up was based on clinical assessment; no postoperative CT or MRI was performed, and no postoperative endoscopic photographs were available. No recurrence of the frontal swelling or periorbital inflammatory symptoms was documented during routine follow-up visits with her primary care physician over the subsequent year.

## Discussion

This case illustrates how a frontal sinus mucocele can mimic a superficial cutaneous abscess, resulting in delayed diagnosis and initial management that does not address the underlying sinus disease. The fluctuating course and transient responses to earlier antibiotic treatments were important clinical clues because antibiotics may reduce secondary infection and surrounding inflammation but do not restore frontal sinus drainage. The failure of the most recent clindamycin course was consistent with the subsequent isolation of clindamycin-resistant *Streptococcus anginosus*. Anteriorly expanding frontal mucoceles may therefore remain unrecognized until progressive expansion and bony remodeling produce a visible forehead mass [1,2,3].

Clinical findings supported periorbital, or preseptal, cellulitis rather than orbital cellulitis: ocular motility was preserved, with no diplopia or visual impairment. In contrast, restricted or painful eye movements, proptosis, or reduced vision should raise concern for postseptal orbital involvement [6]. Nevertheless, periorbital inflammation associated with a frontal lesion should prompt evaluation for underlying sinonasal disease. A delayed presentation of frontal mucopyocele with recurrent periorbital cellulitis has previously been described, illustrating how this condition may be mistaken for a superficial infectious process [7].

The clear, nonpurulent fluid obtained during the initial incision was an important clue that the lesion was not a simple cutaneous abscess. Because the fluid was not analyzed, its exact nature could not be established, and a cerebrospinal fluid component could not be formally excluded given the posterior table erosion and proximity to the dura mater. However, no intraoperative cerebrospinal fluid leak was documented, and no skull base repair or reconstruction was performed. The initially drained fluid should therefore not be interpreted as a confirmed cerebrospinal fluid leak. CT and MRI provided complementary information. CT demonstrated erosion of the anterior and posterior frontal sinus tables and defined the bony anatomy, whereas MRI better characterized the lesion and its relationship to the orbit, periorbita, dura mater, and intracranial compartment [4,5].

Management of a frontal sinus mucocele aims to re-establish sinus drainage and achieve adequate marsupialization. Endoscopic management can provide effective drainage in appropriately selected patients while avoiding the morbidity of an external approach [8]. The surgical approach depends on the extent of the lesion and associated orbital involvement [9]. A Draf IIc approach provides unilateral frontal sinus access that extends across the superior nasal septum toward the contralateral side, providing wider exposure of the frontal sinus drainage pathway in selected unilateral disease [10]. In this patient, endoscopic marsupialization combined with antimicrobial therapy resulted in complete clinical resolution without documented recurrence during one year of clinical follow-up, although long-term anatomical patency was not assessed by postoperative endoscopy or imaging.

Pott’s puffy tumor, defined as frontal bone osteomyelitis associated with a subperiosteal abscess, was considered because of the frontal swelling and erosion of both frontal sinus tables [11]. However, imaging showed an expansile lesion centered within the frontal sinus, without a separately identifiable subperiosteal abscess or specific radiological evidence of osteomyelitis. In addition, the pathology report did not describe histological features of osteomyelitis in the bone fragments examined. These findings favored an infected frontal sinus mucocele with bony erosion, although focal osteomyelitis could not be excluded because no targeted bone biopsy was performed.

This case emphasizes that recurrent frontal swelling, particularly when drainage yields clear rather than purulent fluid or when symptoms recur despite antibiotic therapy, should prompt CT or MRI before further incision to exclude underlying frontal sinus pathology. Early recognition and specialist referral may reduce the risk of potentially serious orbital or intracranial complications.

## Conclusions

Recurrent frontal swelling that improves only transiently with antibiotics or yields clear, nonpurulent fluid should prompt CT or MRI before any further incision. The absence of fever, headache, neurological symptoms, diplopia, or visual impairment does not exclude an infected frontal sinus mucocele with substantial bony erosion. Early recognition and specialist referral are important for avoiding inappropriate drainage and potentially serious orbital or intracranial complications. In this patient, endoscopic marsupialization combined with antimicrobial therapy resulted in complete clinical resolution without documented recurrence during one year of clinical follow-up.
